# Identification and Functional Validation of *ACSL1* and *FABP3* as Muscle-Related Genes Screened by Transcriptomics in Crossbred Duroc × Berkshire × Diannan Small-Eared Pigs

**DOI:** 10.3390/genes16050520

**Published:** 2025-04-29

**Authors:** Bohe Chen, Sui Liufu, Sheng Wen, Kaiming Wang, Wenwu Chen, Lanlin Xiao, Xiaolin Liu, Lei Yi, Jingwen Liu, Xin Xu, Caihong Liu, Wu Wen, Haiming Ma, Qiuchun Deng

**Affiliations:** Yuelushan Laboratory and Key Laboratory of Livestock and Poultry Resources Evaluation and Utilization, Ministry of Agriculture and Rural Affairs, Hunan Agricultural University, Changsha 410128, China; chenhe0213914@163.com (B.C.); liufusui0816@163.com (S.L.); 15885463786@163.com (S.W.); m15116529648@163.com (K.W.); cww1242646778@163.com (W.C.); finafantacy@sina.com (L.X.); lxl810711@163.com (X.L.); ylasoi@163.com (L.Y.); 15093946542@163.com (J.L.); xuxin141596@163.com (X.X.); 17679179352@163.com (C.L.); ww15979545900@163.com (W.W.); mahaiming@huanau.edu.cn (H.M.)

**Keywords:** transcriptomics, local pig breed, pig breeding, *FABP3*, *ACSL1*

## Abstract

**Background:** Crossbreeding strategies that combine the growth performance of Western pig breeds with the meat quality traits of Chinese indigenous breeds have garnered considerable interest. Duroc pigs are known for their high growth efficiency but have relatively low intramuscular fat (IMF) content. In contrast, native breeds like the Diannan Small-Eared pig exhibit superior pork quality with higher IMF levels. This study aimed to compare the muscle growth characteristics and molecular mechanisms between Duroc × Landrace × Yorkshire (DLY) and Duroc × Berkshire × Diannan Small-Eared (DBD) pigs. **Methods:** The longissimus dorsi tissue of 210-day-old DLY and DBD pigs was collected for analysis. HE staining assessed muscle fiber characteristics, IMF content was measured, and ELISA quantified muscle-derived growth and development-related factors. Transcriptome sequencing was conducted, followed by differential gene expression analysis, Gene Ontology (GO), Kyoto Encyclopedia of Genes and Genomes (KEGG), and protein–protein interaction (PPI) analyses. Functional validation of key genes was performed in C2C12 cells. **Results:** DBD pigs exhibited significantly larger muscle fiber diameter and higher IMF content compared to DLY pigs. IGF1 and GH levels were elevated in DBD pigs. Transcriptome analysis identified 185 upregulated and 102 downregulated genes, with enrichment in pathways including PI3K-Akt, MAPK, FoxO, and cGMP-PKG signaling. *ACSL1* and *FABP3* were functionally validated, showing promotion of differentiation and inhibition of proliferation in C2C12 cells. **Conclusions:** DBD pigs exhibit superior muscle growth traits and higher IMF content compared to DLY pigs. *ACSL1* and *FABP3* may serve as key regulators of muscle development in pigs.

## 1. Introduction

As one of the primary sources of meat and livestock products, pork plays a crucial role in daily human life [1]. Lean meat yield is emphasized in high-efficiency pig production systems due to its economic advantages and growing consumer demand [2]. In addition, the sensory properties of pork, including texture and flavor, are key factors influencing consumer choices. IMF refers to the fat within muscle fibers, and its content directly influences the flavor, juiciness, and tenderness of the meat [3,4,5]. Muscle fiber type and fiber diameter are closely linked, and are both known to influence meat quality [6,7,8]. Furthermore, greater proportions of oxidative muscle fibers are generally associated with a higher meat quality; pork rich in oxidative muscle fibers is typically more tender and has a better color [9]. High myosin heavy chains (MyHC) I content leads to an increase in free amino acid levels in muscles, resulting in a richer flavor in the meat, while meat composed of fast-twitch muscle fibers has a less favorable flavor [10]. Multiple reports suggest that variations in muscle fiber composition may influence intramuscular fat deposition, thereby impacting the overall quality attributes of pork [6,11,12,13]. Therefore, exploring the mechanisms underlying muscle growth and development is crucial for improving meat quality and enhancing production efficiency.

The DLY hybrid is known for its superior lean meat production, rapid growth rate, and efficient feed utilization, securing a major presence in both Chinese and global markets [14,15]. Due to long-standing natural and selective breeding practices, China also has many indigenous breeds, which typically exhibit a higher IMF content, greater resilience, and superior meat quality traits compared to genetically improved commercial pigs [2,16,17]. Diannan Small-Eared (DSE) is a breed indigenous to China, and its meat demonstrates better quality and fat deposition than Western breeds. Over a prolonged period of natural selection, it has adapted to the hot, humid subtropical climate. Due to its phenotypic diversity and excellent commercial traits, DSE is a valuable genetic resource deserving of scientific protection and effective utilization [18,19]. Both Berkshire pigs and Duroc pigs are breeds characterized by their high lean meat yield and low fat content. Using Duroc pigs as the sire offers advantages over traditional Berkshire pigs, including faster growth, a higher IMF content, and a higher lean meat yield [20]. To better exploit the superior genetic traits of the DSE and address the gap in optimizing its hybrid progeny, this study used DSE, Duroc, and Berkshire pigs as parents for crossbreeding to obtain the DBD hybrid combination. Through transcriptome sequencing and a comparative analysis of DBD and DLY pigs, this study aims to investigate growth performance and meat quality, and to identify genes responsible for muscle growth and meat quality regulation, offering molecular insights for enhancing local breeds.

## 2. Materials and Methods

### 2.1. Animal Materials

The DBD pigs were sourced from the LanCang Black Pig Trait Measurement Station in Yunnan Province, while the DLY pigs were obtained from the same batch bred at the same time by Tangrenshen Group Co., Ltd. (Changsha, China). At birth, full-sibling piglets with similar body weights were selected from both breeds. The selected DLY (birth weight: 1.38 ± 0.15 kg) and DBD pigs (birth weight: 1.43 ± 0.12 kg) showed no significant differences in birth weight. All pigs were raised under identical nutritional standards and management conditions, and their growth was continuously monitored from birth to slaughter. From 1 to 60 days of age, all pigs were fed the same diet twice daily, consisting of piglet feed containing 18–19% crude protein, at least 1.2% lysine, 0.20–0.40% sodium chloride, 1.8–2% calcium hydrogen phosphate, and 3300–3400 kJ/kg of digestible energy. From 60 to 210 days of age, pigs continued to receive the same diet twice daily, now composed of compound feed containing 14–16% crude protein, 0.25–0.60% sodium chloride, 0.60–1.50% calcium, and 3100–3200 kJ/kg of digestible energy, with water provided ad libitum. Longissimus dorsi muscle samples were collected from 6 DBD pigs and 6 DLY pigs at 210 days of age. The longissimus dorsi muscle samples were stored in cryotubes for freezing. Following blood collection, the samples were kept at 25 °C for 1 h, and then centrifuged at 4 °C, 3000 rpm for 5 min. The upper layer was harvested and preserved at −80°C.

### 2.2. Section Preparation and HE Staining

Muscle samples were cut into 1 cm^3^ pieces along the direction of the muscle fibers, fixed in 4% paraformaldehyde for 24 h, followed by dehydration, clearing, paraffin embedding, and sectioning. After staining with Hematoxylin and Eosin (HE), the sections were observed and photographed.

### 2.3. ELISA

ELISA kits for porcine insulin-like growth factor 1 (ELK5724, ELK Biotechnology, Wuhan, China) and porcine growth hormone (ELK5721, ELK Biotechnology, Wuhan, China) were used to analyze the longissimus dorsi muscle samples, following the protocols outlined in the respective kit manuals.

### 2.4. Cell Culture

C2C12 (ATCC, New York, NY, USA) was cultivated using high-glucose DMEM (Gibco, Waltham, MA, USA), and 10% FBS (Gibco, Waltham, MA, USA) and 1% pen–strep mixture (Gibco, Waltham, MA, USA) were added. The mixture was then placed in a 37 °C environment containing 5% CO_2_. For myogenic differentiation, C2C12 cells received DMEM containing 2% HBS (Gibco, Waltham, MA, USA) once they reached 80% confluence. The overexpression and interference of *ACSL1* and *FABP3*, along with their respective controls, were sourced from GenePharma Corporation (Suzhou, China), with the siRNA sequences listed in Table 1. After the C2C12 cells reached 80% confluence, they were seeded into a 6-well plate. For proliferation assays, transfection of C2C12 was performed with Lipofectamine 2000 (Invitrogen, Waltham, MA, USA). When these cells reached 50% confluence, the growth medium was refreshed, and the cells were further incubated for 6 h. For differentiation assays, the cells were transfected with Lipofectamine 2000 when they reached 80% confluence. After 6 h, differentiation medium was added to replace the previous medium.

### 2.5. RNA Extraction and Quantitative PCR Analysis

RNA extraction was performed using TRIzol reagent (Invitrogen, Waltham, MA, USA), followed by storage at −80 °C. The RNA was then reverse-transcribed to cDNA using the TAKARA^TM^ PrimeScript^TM^ Reverse Transcription Kit (Takara Bio Inc., Kusatsu, Japan). Quantitative PCR was performed with the SYBR Green Kit (TransGen, Beijing, China). Gene expression was quantified using the 2^−ΔΔCt^ method with *GAPDH* as the endogenous control. Primers were designed using Primer premier 6.0, and the sequences of all the primers are provided in Table 2.

### 2.6. Cell Proliferation Analysis via CCK-8 Kit

C2C12 cells were seeded into 96-well plates. The cells were transfected when the confluence reached 30%. After incubating at 37 °C for 0, 12, 24, 36, and 48 h, the cells were treated with culture medium containing ten percent CCK8 reagent (APExBIO, Houston, TX, USA), followed by 4 h incubation at 37 °C. A spectrometer (Molecular Devices, San Francisco, CA, USA) was used to measure protein absorption peaks.

### 2.7. EdU Analysis

The existing medium was exchanged for fresh solution supplemented with EdU when the transfected C2C12 cells attained 70–80% confluency, followed by incubation at 37 °C for 2 h. Cell proliferation analysis was conducted with the Alexa Fluor 555 detection kit (Meilun, Dalian, China) through EdU incorporation assays. Fluorescence imaging was conducted using the Carl Zeiss LSM800 (Carl Zeiss AG, Oberkochen, Germany), followed by quantitative analysis of the captured images.

### 2.8. Fluorescent Immunolabeling

C2C12 cells were fixed with 4% paraformaldehyde for 30 min. After treatment with 0.5% Triton X-100 for 20 min, non-specific binding was blocked using 5% BSA for 2 h. The cells were then incubated with a primary antibody overnight at 4 °C. A secondary antibody was added and the mixture was incubated for 2 h at 25 °C in the dark. Nuclei were counterstained with DAPI for 10 min. Fluorescence was preserved with anti-fade mounting medium, and images were acquired using a Leica SP8 confocal microscope (Leica, Wetzlar, Germany).

### 2.9. Western Blot Assay

Total protein was extracted using Abiowell RIPA lysis buffer (Abiowell, Changsha, China). Protein concentration was measured using the Abiowell BCA protein detection kit (Abiowell, Changsha, China), and protein blotting was performed according to the standard protocol. The following is the antibody information:

GAPDH 1:1000 (ET1601-4, Mouse, HUABIO, Hangzhou, China)

PCNA 1:2000 (AF02986, Rabbit, AFBio, Changsha, China)

CDK4 1:2000 (AF06640, Rabbit, AFBio, Changsha, China)

CCND2 1:1000 (ER63155, Rabbit, HUABIO, Hangzhou, China)

MYOD 1:1000 (ER63155, Rabbit, HUABIO, Hangzhou, China)

MyoG 1:1000 (AB2146602, Rabbit, DSHB, Iowa, IA, USA)

MEF2C 1:1000 (10056-1-AP, Rabbit, Sanying, Wuhan, China)

## 3. Results

### 3.1. Morphological Variations and ELISA Results

**Figure 1:**
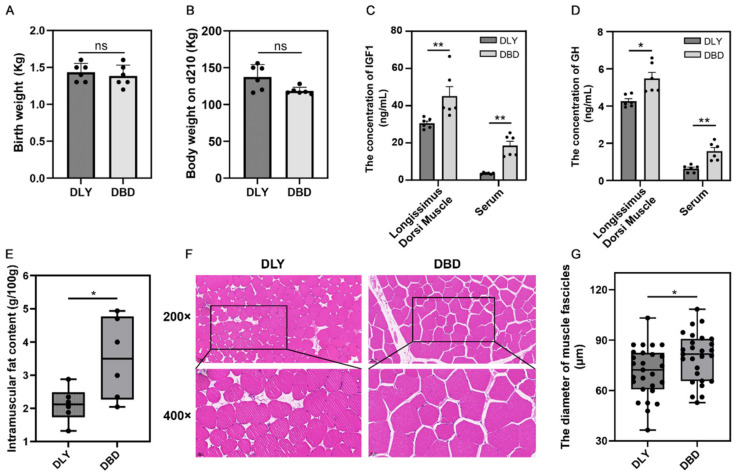
Birth weight, slaughter weight, and comparative analysis of morphological differences in muscle tissue and ELISA results in longissimus dorsi muscle: (**A**) Birth weight of DLY and DBD. (**B**) Body weight at 210 days of age. (**C**,**D**) Comparison of IGF1 and GH levels. (**E**) Comparison of muscle fiber diameter. (**F**) HE staining of muscle samples. (**G**) Comparison of intramuscular fat content. * Represents a significant difference (*p* < 0.05); ** represents an extremely significant difference (*p* < 0.01). “ns” represents no significant difference (*p* < 0.05).

The birth weights and 210-day-old weights of DBD (*n* = 6) and DLY (*n* = 6) pigs are shown in Figure 1A,B. The ELISA results showed significantly higher IGF1 and GH concentrations in the DBD (*n* = 6) group compared to the DLY (*n* = 6) group (*p* < 0.05 or 0.01, Figure 1C,D). The morphological analysis revealed that the longissimus dorsi muscle of 210-day-old DBD pigs (*n* = 6) exhibited significantly greater muscle fiber diameters and intramuscular fat (IMF) content compared to DLY pigs (*n* = 6) (*p* < 0.05, Figure 1E–G). Therefore, RNA-seq was used to investigate the genes associated with longissimus dorsi muscle in pigs. Transcriptome sequencing was performed on DLY (*n* = 6) and DBD (*n* = 6) pigs, yielding an average of 42,000,000 clean reads. The Q30 base percentage exceeded 95.08%. After obtaining the clean reads, we aligned the reads from both the DLY and DBD groups, with mapping rates ranging from 96.59% to 96.88% (Table 3).

### 3.2. DEGs in the Longissimus Dorsi Muscle of DLY and DBD

**Figure 2:**
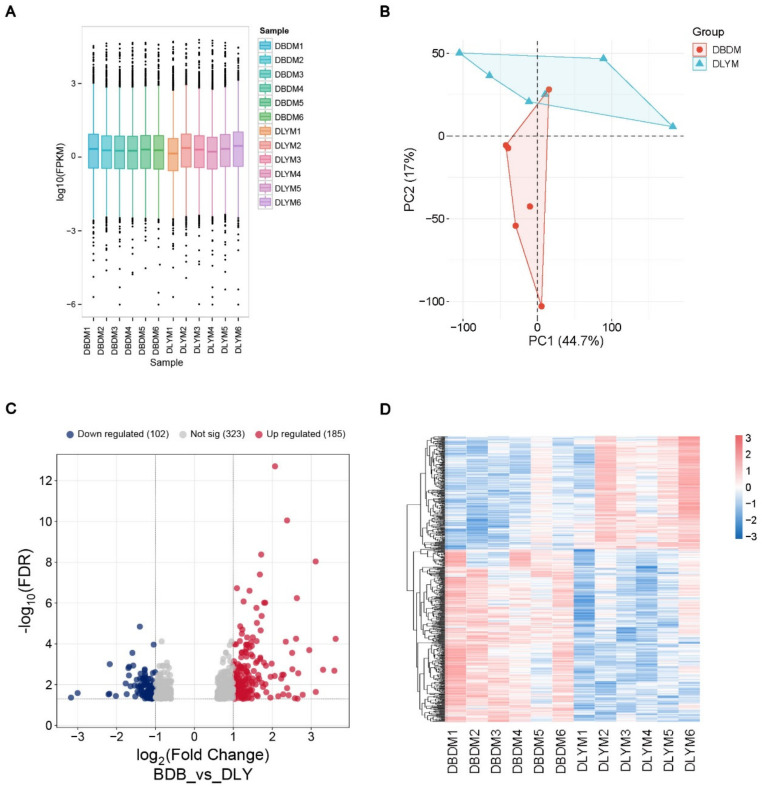
Analysis of DEGs: (**A**) Box plot depicting mRNA expression patterns. (**B**) PCA plot of all samples. (**C**) Volcano plot (threshold for DEGs is FDR < 0.05, |log2 (FC)| > 1). (**D**) Heatmap.

The box plot (Figure 2A) illustrates the distribution of gene expression levels between DBD and DLY groups, revealing discernible intra- and inter-group variations. The PCA plot shows that the data from the two groups were relatively easy to distinguish, further confirming the high quality of the RNA-seq data (Figure 2B). The volcano plot (Figure 2C) further delineates differential gene expression, identifying 185 upregulated and 102 downregulated genes (Table S1). The heatmap displays the homogeneous clustering of gene expression patterns within each group and marked divergence between groups (Figure 2D).

### 3.3. GO Enrichment

**Figure 3:**
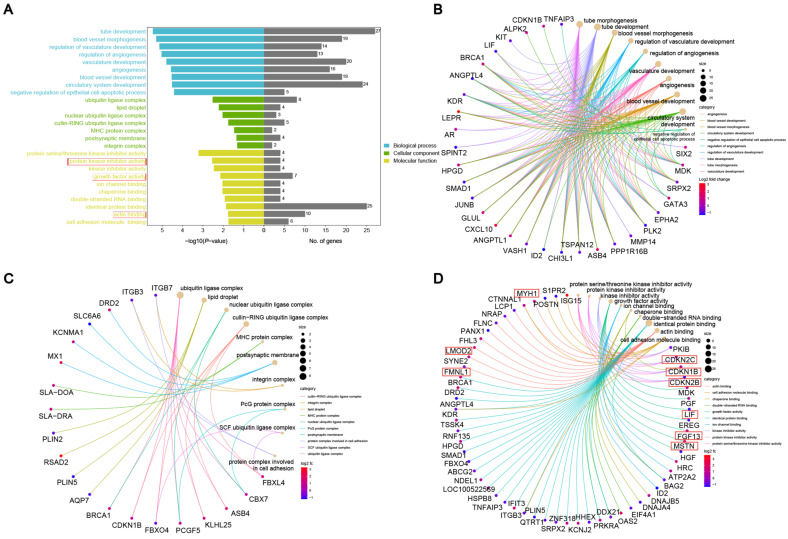
GO annotation and enrichment analysis of DEGs: (**A**) GO functional categorization. (**B**) Enriched biological processes. (**C**) Cellular components. (**D**) Molecular functions. The red boxes represents muscle related.

A functional enrichment analysis was conducted on the targeted DEGs to identify GO-enriched terms contributing to muscle formation and growth. The genes were grouped into three primary GO classifications: biological process (BP), cellular component (CC), and molecular function (MF) (Figure 3A). The biological process category includes muscle cell differentiation, myotube differentiation, muscle cell development, and other muscle-related terms. In the cellular component category, terms, such as ubiquitin ligase complex, lipid droplet, integrin complex, postsynaptic membrane, and cullin–RING ubiquitin ligase complex, are involved in muscle development, muscle contraction, and intramuscular lipid storage metabolism. In the molecular function category, terms, such as protein kinase inhibitor activity, growth factor activity, and actin binding, are related to muscles, including genes, such as *CDKN1B*, *CDKN2B*, *CDKN2C*, *LIF*, *FGF13*, *FMNL1*, *LMOD2*, and *MYH1* (Table 4).

### 3.4. KEGG Enrichment Analysis and qRT-PCR Validation

**Figure 4:**
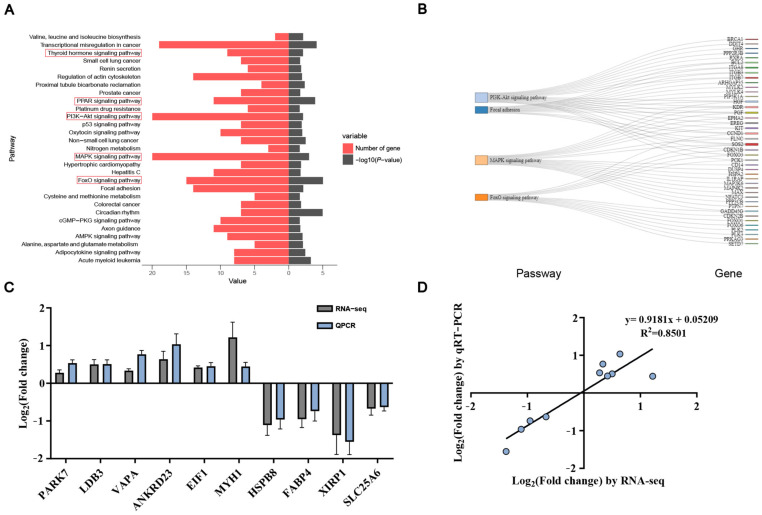
KEGG and qRT-PCR confirmation of RNA-seq data: (**A**) KEGG analysis results. (**B**) DEGs associated with muscle-related pathways. (**C**) Verification of transcriptome sequencing results. (**D**) Linear regression analysis of expression levels between RNA-seq and qRT-PCR data. The *x*-axis represents log 2FC values from RNA-seq, and the *y*-axis represents log 2FC values from qRT-PCR. The red boxes represents muscle related.

Differentially expressed genes were analyzed for pathway enrichment using the KEGG database. The thyroid hormone, PPAR-γ, PIK3-Akt, MAPK, and FoxO pathways are likely to play significant roles in muscle development (Figure 4A). In total, 56 genes were assigned to the five pathways (Figure 4B). To confirm the reliability of the RNA-seq results, 10 differentially expressed genes (*PARK7*, *LDB3*, *VAPA*, *ANKRD23*, *EIF1*, *MYH1*, *HSPB8*, *FABP4*, *XIRP1*, and *SLC25A6*) were randomly selected for qPCR, which revealed that the expression trends of the screened genes were highly consistent with the RNA-seq data (Figure 4C). A strong positive correlation (R^2^ = 0.8501; Figure 4D) was observed between the two methods, further validating the reliability of the DEGs identified through transcriptomic sequencing.

### 3.5. Protein–Protein Interaction Network Analysis and Identification of Hub Genes

**Figure 5:**
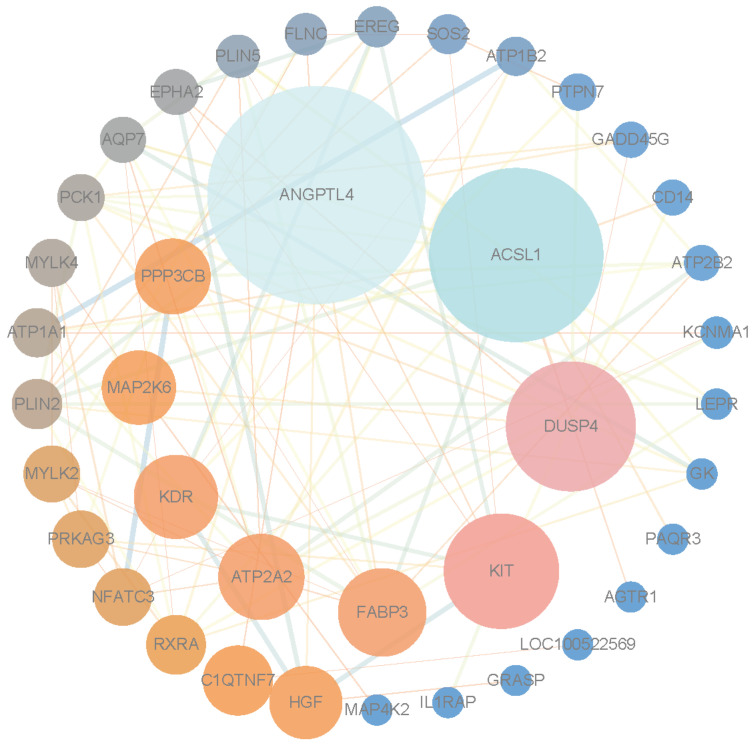
Protein–protein interaction network.

The DEGs in the regulatory network were analyzed using STRING, generating a protein–protein interaction network (Figure 5). The results revealed that *ANGPTL4*, *ACSL1*, *DUSP4*, *KIT*, and *FABP3* were the top five genes. *ACSL1* and *FABP3* were selected for functional validation in C2C12 myoblasts.

### 3.6. ACSL1 Inhibits the Proliferation of C2C12

**Figure 6:**
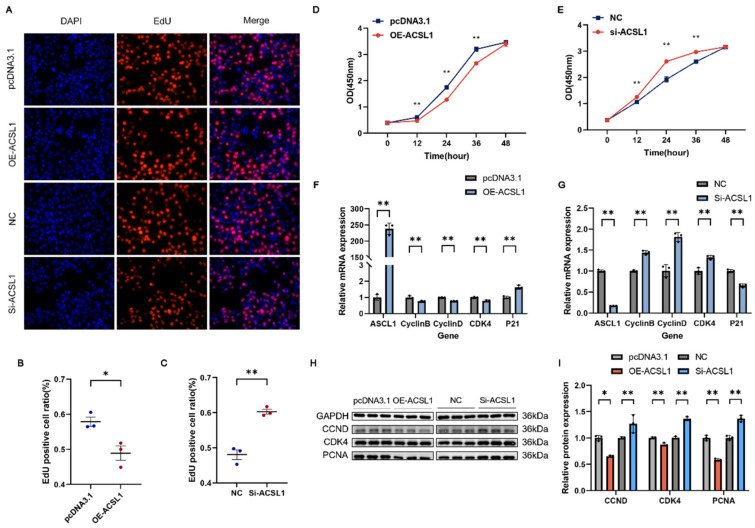
*ACSL1* suppresses myoblast proliferation: (**A**) Representative images of EdU assay after transfection with *ACSL1* interference or overexpression vectors (400×). (**B**,**C**) EdU-positive cell rate. (**D**,**E**) Proliferation of transfected C2C12 cells was assessed using CCK-8 assay. (**F**–**I**) qRT-PCR and Western blot analysis following transfection with *ACSL1* interference RNA and overexpression vectors. Results are expressed as mean ± SEM (*n* = 3). * Represents a significant difference (*p* < 0.05); ** represents an extremely significant difference (*p* < 0.01).

To investigate the role of *ACSL1* in myoblast proliferation, we conducted experiments involving *ACSL1* overexpression or interference. The EdU analysis revealed a significant reduction in myoblast proliferation due to *ACSL1* overexpression (Figure 6A,B). In contrast, the Si-ACSL1 group had markedly enhanced proliferation (Figure 6A,C). C2C12 proliferation was significantly suppressed in the *ACSL1* overexpression group, as confirmed by CCK-8 (Figure 6D). Conversely, *ACSL1* interference significantly enhanced myoblast proliferation (Figure 6E). After 24 h of transfection, *ACSL1* overexpression led to a significant decrease in *CyclinB*, *CyclinD*, and *CDK4* mRNA levels, as confirmed by qRT-PCR analysis, while increasing the mRNA level of *P21*. Western blot showed a significant increase in protein expression was found for CCND, CDK4, and PCNA. The *ACSL1* interference results were opposite to those from *ACSL1* overexpression (Figure 6F–I). These findings indicate that *ACSL1* inhibits myoblast proliferation.

### 3.7. FABP3 Inhibits the Proliferation of C2C12

**Figure 7:**
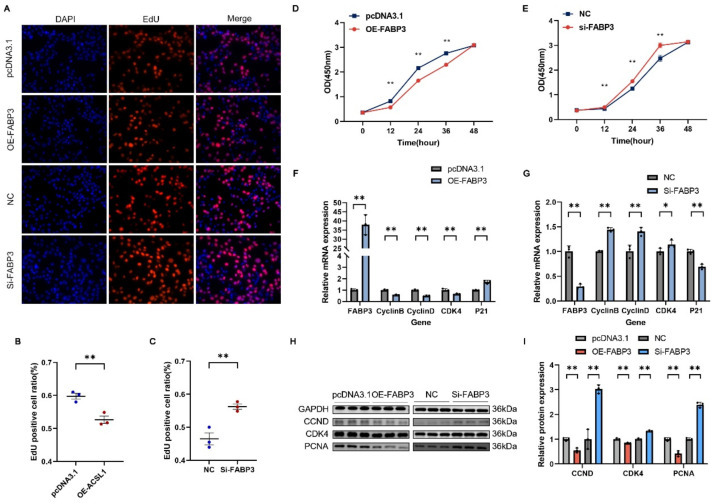
*FABP3* suppresses myoblast proliferation. (**A**) Representative images of EdU assay following transfection with *FABP3* knockdown or overexpression vectors (400×). (**B**,**C**) Rate of EdU-positive cells. (**D**,**E**) Myoblast proliferation in transfected C2C12 cells was evaluated using CCK-8 assay. (**F**–**I**) qRT-PCR and Western blot analysis after transfection with *FABP3* knockdown RNA and overexpression constructs. Results are presented as mean ± SEM (*n* = 3). * Represents a significant difference (*p* < 0.05); ** represents an extremely significant difference (*p* < 0.01).

We also constructed the *FABP3* overexpression vector and designed siRNA targeting *FABP3* to knock down its expression. The EdU and CCK8 assays showed that *FABP3* upregulation markedly enhanced the proliferation of myoblasts (Figure 7A,B,D), while silencing *FABP3* expression markedly suppressed proliferation (Figure 7A,C,E). qRT-PCR analysis revealed that the mRNA levels of *CyclinB*, *CyclinD*, and *CDK4* were notably reduced by *FABP3* overexpression after 24 h of transfection, while *P21* mRNA expression was significantly upregulated (Figure 7F). The Western blot analysis showed that *FABP3* overexpression led to a significant elevation in the protein expression of CCND, CDK4, and PCNA (Figure 7H,I). In contrast, interference with *FABP3* resulted in opposite effects compared to *FABP3* overexpression (Figure 7G–I).

### 3.8. ACSL1 Facilitates the Differentiation of C2C12

**Figure 8:**
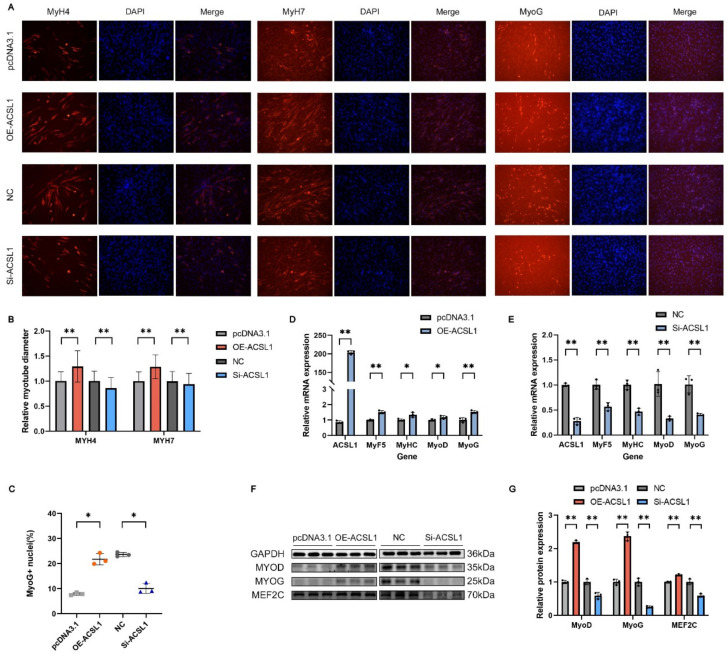
*ACSL1* promotes myoblast differentiation: (**A**) Immunofluorescence staining of myoblast differentiation after *ACSL1* silencing and overexpression (200×). (**B**,**C**) Muscle fiber diameter and proportion of MyoG-positive cell nuclei. (**D**–**G**) qRT-PCR and Western blot analyses of differentiation marker gene expression after silencing and overexpressing *ACSL1*. Results are shown as mean ± SEM (*n* = 3). * Represents a significant difference (*p* < 0.05); ** represents an extremely significant difference (*p* < 0.01).

We assessed the impact of *ACSL1* interference and overexpression on myoblast differentiation. The transfection of *ACSL1* overexpression vectors significantly upregulated the mRNA expression levels of both *ACSL1* and myogenic differentiation markers *MyoD*, *MyoG*, *MyHC* and *MyF5*, as well as MyoD, MyoG, and MEF2C protein levels at four days post-transfection (Figure 8D,F,G). The immunofluorescence analysis of MyH4, MyH7, and MyoG protein expression at day six of differentiation revealed that OE-ACSL1 influenced the muscle fiber diameter in MyH4- and *MyH7*-positive cells and the MyoG-positive cell nuclei rate (Figure 8A–C). In contrast, *ACSL1* silencing resulted in the opposite effects (Figure 8A–C,E–G). These findings suggest that *ACSL1* promotes myoblast differentiation.

### 3.9. FABP3 Promotes the Differentiation of C2C12

**Figure 9:**
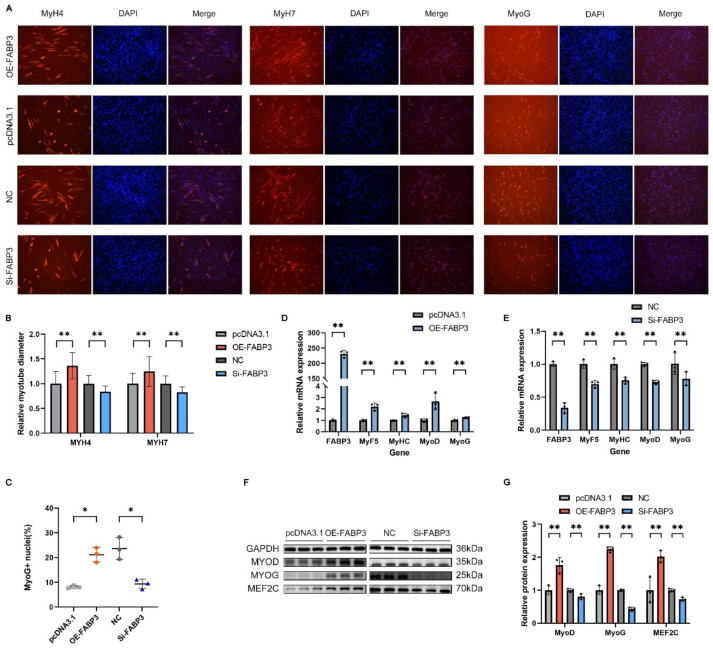
*FABP3* promotes myoblast differentiation: (**A**) Immunofluorescence staining of myoblast differentiation after *FABP3* silencing and overexpression (200×). (**B**,**C**) Muscle fiber diameter and proportion of MyoG-positive cell nuclei. (**D**–**G**) After silencing and overexpressing *FABP3*, qRT-PCR and Western blot analyses of differentiation-related gene expression. Results are shown as mean ± SEM (*n* = 3). * Represents a significant difference (*p* < 0.05); ** represents an extremely significant difference (*p* < 0.01).

We also investigated the impact of changes in *FABP3* gene expression on myoblast differentiation. The results demonstrated that after four days of *FABP3* overexpression, the mRNA levels of *FABP3* and differentiation marker genes *MyoD*, *MyoG*, *MyHC*, and *MyF5* were significantly increased, with a corresponding significant rise in the protein expression of MyoD, MyoG, and MEF2C (Figure 9D,F,G). The immunofluorescence analysis of MyH4, MyH7, and MyoG protein expression at day six of differentiation revealed that *FABP3* overexpression significantly increased the muscle fiber diameter of *MyH4*- and MyH7-positive cells and the proportion of MyoG-positive cell nuclei (Figure 9A–C). Conversely, the mRNA and protein levels of these genes were significantly reduced when *FABP3* expression was silenced. In the immunofluorescence assays, the Si-FABP3-treated group exhibited smaller muscle fiber diameters for MyH4- and MyH7-positive cells and a reduced proportion of MyoG-positive cell nuclei compared to the control group (Figure 9A–C,E–G). These results suggest that *FABP3* promotes myoblast differentiation.

## 4. Discussion

Currently, research on the genetic mechanisms of hybrid vigor in pigs is still underdeveloped. While hybrid vigor and three-way cross-pig breeds are widely applied in animal husbandry, the underlying genetic and molecular mechanisms remain insufficiently understood. Moreover, technologies for predicting and stabilizing hybrid vigor are still in the developmental stage, which hinders its precise and efficient use. DBD pigs exhibit a high IMF content and growth potential. Therefore, uncovering the genetic and molecular mechanisms underlying these advantageous traits will help address the systemic weaknesses in hybrid pig populations and provide a theoretical foundation for developing breakthrough breeds.

In this study, we compared the phenotypic traits of the longissimus dorsi muscle between DBD and DLY pigs. The results showed that the DBD pigs exhibited greater muscle fiber diameters and a higher IMF content than the DLY pigs. The ELISA kits revealed that the DBD pigs had higher concentrations of IGF1 and GH in their longissimus dorsi muscle. The current research indicates that both muscle and IMF growth and development influence meat quality. Muscle growth primarily affects water-holding capacity and tenderness. Higher muscle fiber density results in better water retention and a finer meat texture. Type I muscle fibers have a higher density, which enables the muscle to retain more water per unit volume [29,30,31]. Therefore, pork with enriched Type I muscle fibers tends to retain water more effectively. A substantial body of research indicates that intramuscular fat influences multiple attributes of meat quality, such as tenderness, flavor, juiciness, and nutritional profile. Generally, elevated IMF levels are associated with a superior eating quality [32,33,34,35]. IGF1 and GH are known to be involved in muscle growth. Activation of the GH/IGF-1 signaling pathway facilitates skeletal muscle hypertrophy [36,37]. The higher levels of IGF-1 and GH in DBD pigs support their higher IMF content and suggest that DBD pigs may have greater growth potential. In light of the excellent traits exhibited by DBD pigs, a comparative transcriptomic analysis was conducted between DBD and DLY pigs. Numerous DEGs were detected from both DBD and DLY pigs. These DEGs were enriched in muscle growth and development-related pathways, including thyroid hormone, PIK3-Akt, MAPK, and FoxO. Candidate hub genes were identified through an analysis of the PPI network, and *ACSL1* and *FABP3* were selected for further validation.

ACSL1 is markedly upregulated during porcine preadipocyte differentiation, where it promotes triglyceride accumulation by enhancing the transcription of key adipogenic genes while concurrently repressing lipolytic marker expression [38]. Moreover, ACSL1 appears to operate within the C/EBPα–ACSL1 regulatory axis to modulate adipogenesis [39]. ACSL1 is involved in cardiac lipid metabolism through myocardial fatty acid oxidation and may contribute to the loss of myocardial regenerative potential in mice beginning at seven days after birth [40]. Inhibition of ACSL1 has been shown to significantly enhance myocardial repair following myocardial infarction in mice [41]. Loss of ACSL1 function leads to impaired lipid oxidation across skeletal muscle, myocardium, and adipose tissue [42]. A linear association exists between liver ACSL1 expression and muscle fiber diameter, with higher expression observed in mice possessing larger muscle fibers [43]. Prior research has demonstrated significantly elevated hepatic and longissimus dorsi ACSL1 mRNA levels in Diannan Small-Eared pigs compared to Landrace pigs [44].

The cytoplasmic protein FABP3, known for its lipid transport function, is predominantly present in skeletal and cardiac muscle, involved in intracellular lipid metabolism and signal transduction [45,46], and is crucial for regulating muscle growth and enhancing IMF content [47,48]. It has also been reported that FABP3 has been implicated in several diseases, including cardiovascular diseases [49,50,51], diabetic nephropathy [52], cancer [53], and Alzheimer’s disease [54,55]. FABP3 can stimulate glucose uptake by promoting the AMPK-dependent phosphorylation of AS160 in skeletal muscle. Additionally, FABP3 may enhance AS160 phosphorylation by maintaining insulin-stimulated Akt activity under lipotoxic conditions [56].

In this study, EdU staining and CCK-8 assays revealed that the overexpression and knockdown of *ACSL1* and *FABP3* inhibited and promoted C2C12 myoblast proliferation, respectively. The RT-qPCR and Western blot analyses showed significant changes in the mRNA and protein expression levels of proliferation marker genes. The kinase CDK4 regulates the G1 to S phase transition, and cyclins (including CyclinB and CyclinD) orchestrate various cell cycle stages through complex formation with cyclin-dependent kinases, which in turn modulate cell proliferation. PCNA expression promotes fibroblast proliferation. CyclinB, CyclinD, CDK4, and PCNA act as positive regulators of cell proliferation [57,58]. p21 is a cyclin-dependent kinase inhibitor that plays a key role in mediating G1 phase cell cycle arrest [59]. After overexpressing and silencing *ACSL1* and *FABP3*, the results of RT-qPCR and Western blotting were consistent with those reported in previous studies.

During myogenesis, myogenic cells withdraw from the cell cycle and initiate differentiation, resulting in an inverse relationship between proliferation and differentiation. MyHC acts as the molecular motor in muscle contraction, forming the structural framework of the myofibril thick filaments. Different MyHC isoforms correspond to distinct muscle fiber types and modulate their physiological properties, making them reliable markers for late-stage myotube differentiation [60]. MyoD is a key regulatory factor in myogenic differentiation. It is expressed at low levels in undifferentiated myoblasts, with its expression increasing during the early stages of differentiation. As a transcription factor, MyoD upregulates the expression of genes like MyoG and MEF2C. MyoG regulates the terminal differentiation, fiber size, maturation, and expression of certain MRFs in myogenic cells. MyoG deficiency decreases the expression of MyF5. The process of muscle cell differentiation is modulated by the MEF2 family, which binds to specific DNA sequences in muscle-related gene promoters, facilitating the upregulation of MyoD and MyoG [61]. In conclusion, MYOD, MYOG, MyF5, MyHC, and MEF2C are all key factors that promote myogenic differentiation. After overexpressing and interfering with *ACSL1* and *FABP3*, we quantified the expression of these differentiation markers in myoblast cells. The results demonstrated that *ACSL1* and *FABP3* promote C2C12 cell differentiation, as confirmed by immunofluorescence experiments. Overall, *ACSL1* and *FABP3* inhibit C2C12 myoblast proliferation and promote their differentiation.

According to the results of the above studies, our findings show that DBD pigs have larger muscle fiber diameters and a higher intramuscular fat content compared to DLY pigs. Additionally, IGF1 and GH concentrations are increased in muscle tissue in DBD pigs. *ACSL1* and *FABP3* are crucial in regulating the growth and differentiation of myogenic cells. These results hold substantial implications for pig breeding and meat quality enhancement, and understanding the molecular mechanisms of muscle growth. However, the molecular mechanisms behind the genes and pathways regulating muscle growth, as well as their impacts and mechanisms at the individual level, still require further in-depth research.

## 5. Conclusions

A transcriptomic analysis was performed to compare the differences between DLY pigs and DBD pigs. The muscle fiber diameter, IMF content, and growth factor levels were measured. The results indicated that a significant number of DEGs were identified in the longissimus dorsi muscles of DBD and DLY pigs. The DEGs were enriched in signaling pathways associated with muscle, such as thyroid hormone, PIK3-Akt, MAPK, and FoxO. The DBD pigs exhibited higher muscle fiber diameters, IMF contents, and growth factor expression levels compared to the DLY pigs. According to the transcriptome analysis, the key DEGs *ACSL1* and *FABP3* were functionally validated at the cellular level. Both genes were found to inhibit C2C12 myoblast proliferation and promote myoblast differentiation. In summary, our study examined the phenotypic differences between DLY pigs and DBD pigs, identified feasible breeding candidate genes, and provided valuable reference breeds and a theoretical foundation for improving pork quality and enhancing local pig breeds.

## Figures and Tables

**Table 1:** Sequences of small interfering RNAs.

Name	Sense	Antisense
Si-FABP3	GGA AGC UAG UGG ACA GCA ATT	UUG CUG UCC ACU AGC UUC CTT
Si-ACSL1	GUG UCA UGG AGC UAA GAU ATT	UAU CUU AGC UCC AUG ACA CTT
Si-NC	UUC UCC GAA CGU GUC ACG UTT	ACG UGA CAC GUU CGG AGA ATT

**Table 2:** Primers applied for quantitative PCR.

Target Gene	Oligonucleotide Sequence	Annealing Temp (°C)	Product Size (bp)	GeneBank No.
*EIF1*	F: AATTTGCCTGCAATGGCACT	59	130	XM_003482982. 4
	R: TTCAGCTGGTCGTCCTTAGC			
*HSPB8*	F: TCCTGCTGCTTCTCCTCGTGTT	63	79	XM_001929585. 5
	R: TTAAGCCGGAGGAGCTGATGGT			
*MYH1*	F: AACCTCTCCAAGTTCCGCAAGC	64	68	NM_001104951. 2
	R: TCAGCAATGTCAGCCCGTTCCT			
*VAPA*	F: TGGTGAAGCTGTCGGAGGAGAA	63	136	XM_021096055. 1
	R: AGTGAAGGAAGAGGGCTGGTGA			
*ANKRD23*	F: TGCCTGTGGAGCCCATATTGAC	63	108	NM_001315720. 1
	R: CCATAGAGCAGCAGCACCTTCA			
*PARK7*	F: ATCGGAGTCTGCTGCTGTGAAG	63	138	NM_001078663. 1
	R: AGTGGGTGCGTCGTAACTTTGC			
*LDB3*	F: AGCCCTTCGGGAATAGCCTCTT	64	163	XM_005657425. 3
	R: AAGCAGGTGTCGTGCCAGGTAT			
*FABP4*	F: TGCCTTCAAATTGGGCCAGGAA	64	112	NM_001002817. 1
	R: TCCATCCCACTTCTGCACCTGT			
*SLC25A6*	F: ATCACCAAGTCTGACGGCATCC	63	161	NM_214418. 2
	R: GCGATCATCCAGCTCACCACAA			
*XIRP1*	F: CTGCTGTGACGAGACCTGACTT	63	61	NM_001143928. 1
	R: TGCTGGCTGGAGTCCTCATCAT			
*CyclinB*	F: AACTTCAGCCTGGGTCG	57	249	NM_172301. 3
	R: CAGGGAGTCTTCACTGTAGGA			
*CyclinD*	F: TAGGCCCTCAGCCTCACTC	60	80	NM_001379248. 1
	R: CCACCCCTGGGATAAAGCAC			
*CDK4*	F: CGAGCGTAAGGCTGATGGAT	60	177	NM_001355005. 1
	R: CCAGGCCGCTTAGAAACTGA			
*p21*	F: GAGCAAAGTGTGCCGTTGTC	60	112	NM_001111099. 2
	R: AAAGTTCCACCGTTCTCGGG			
*MyoD*	F: AAGACGACTCTCACGGCTTG	60	169	NM_010866. 2
	R: GCAGGTCTGGTGAGTCGAAA			
*MyoG*	F: CAATGCACTGGAGTTCGGT	58	134	NM_031189. 2
	R: CTGGGAAGGCAACAGACAT			
*MyHC*	F: CAGAAGCAACGAGAGGAG	55	88	XM_017314318. 3
	R: GGTCAGCAGAGTTCAGATT			
*MyF5*	F: CAGGAATGCCATCCGCTACA	60	78	XM_006513319. 3
	R: CCCGGCAGGCTGTAATAGTT			
*ACSL1*	F: CCTGGACGACTTGTTGAA	54	170	NM_001302163. 2
	R: CATCTATCTGCGACCTGAA			
*FABP3*	F: ACCTGGAAGCTAGTGGACAG	59	106	NM_010174. 2
	R: TGATGGTAGTAGGCTTGGTCAT			
*GAPDH*	F: AGGTGGTGAAGCAGGCATCTGA	64	114	NM_001289726. 2
	R: CGGCATCGAAGGTGGAAGAGTG			

**Table 3:** Sequencing basic data of longissimus dorsi muscle tissue in DLY and DBD pigs.

Sample ID	Clean Reads	GC (%)	Q30 (%)	Mapped Reads	Mapping Rate
DLYM1	41,023,660	53.46	95.62	39,655,191	96.66%
DLYM2	43,610,182	52.12	95.61	42,249,383	96.88%
DLYM3	39,986,234	52.84	95.08	38,650,277	96.66%
DLYM4	40,895,390	53.38	95.53	39,504,038	96.60%
DLYM5	40,335,290	52.31	94.58	38,875,712	96.38%
DLYM6	41,858,308	51.59	95.43	40,451,926	96.64%
DBDM1	42,224,020	51.61	95.59	40,808,828	96.65%
DBDM2	41,301,168	51.55	95.55	39,924,198	96.67%
DBDM3	42,115,656	53.14	95.64	40,779,108	96.83%
DBDM4	40,474,448	52.71	95.53	39,095,163	96.59%
DBDM5	43,631,020	52.36	95.74	42,239,474	96.81%
DBDM6	43,820,494	52.07	95.78	42,400,827	96.76%

**Table 4:** Candidate genes related to muscle development.

Gene	Passway	Function	Reference
*CDKN1B*	protein kinase inhibitor activity	Reduced muscle fiber diameter.	[21]
*CDKN2B*	protein kinase inhibitor activity	Involved in TGFβ signaling regulation, impacting smooth muscle cell proliferation and apoptosis.	[22]
*CDKN2C*	protein kinase inhibitor activity	Reduces the expression of cyclin-dependent kinase CDK4, thereby inhibiting the proliferation of muscle cells.	[23]
*LIF*	growth factor activity	Facilitates both the self-renewal and differentiation processes of muscle stem cells.	[24]
*FGF13*	growth factor activity	Upregulates the p27 mRNA level, downregulates the expression of Cyclin E and Spry1 proteins, activates the ERK1/2 signaling pathway, and inhibits myoblast differentiation.	[25]
*FMNL1*	actin binding	Regulates the content of actin.	[26]
*LMOD2*	actin binding	Regulates the sarcomere length in skeletal muscle, and its deficiency results in reduced muscle contractility.	[27]
*MYH1*	actin binding	The myosin heavy chain encoded by MYH1 is a major component of fast-twitch muscle fibers (Type II fibers).	[28]

## Data Availability

The data presented in this study are available on request from the corresponding author due to potential future use in related studies.

## Supplementary Materials

The following supporting information can be downloaded at https://www.mdpi.com/article/10.3390/genes16050520/s1, Table S1: A total of 287 DEGs were identified when comparing DBD to DLY.
